# Mining and unearthing hidden biosynthetic potential

**DOI:** 10.1038/s41467-021-24133-5

**Published:** 2021-06-23

**Authors:** Kirstin Scherlach, Christian Hertweck

**Affiliations:** 1 Department of Biomolecular Chemistry, Leibniz Institute for Natural Product Research and Infection Biology, HKI, Jena, Germany; 2 Faculty of Biological Sciences, Friedrich Schiller University Jena, Jena, Germany

**Keywords:** Computational biology and bioinformatics, Biosynthesis, Natural products, Drug discovery and development

## Abstract

Genetically encoded small molecules (secondary metabolites) play eminent roles in ecological interactions, as pathogenicity factors and as drug leads. Yet, these chemical mediators often evade detection, and the discovery of novel entities is hampered by low production and high rediscovery rates. These limitations may be addressed by genome mining for biosynthetic gene clusters, thereby unveiling cryptic metabolic potential. The development of sophisticated data mining methods and genetic and analytical tools has enabled the discovery of an impressive array of previously overlooked natural products. This review shows the newest developments in the field, highlighting compound discovery from unconventional sources and microbiomes.

## Abstract (web summary)

Natural products are an important source of bioactive compounds and have versatile applications in different fields, but their discovery is challenging. Here, the authors review the recent developments in genome mining for discovery of natural products, focusing on compounds from unconventional microorganisms and microbiomes.

## Introduction

Natural products are an unparalleled source of bioactive compounds, many of which have found application in medicine or agriculture or are important drivers of organismal interactions^1,2^. Traditionally, these compounds were isolated from microbes and plants by bioactivity-guided approaches; however, conventional strategies now fail to cover the constant demand for new chemical entities^3^. Advances in genomics, bioinformatics, and chemical analytics have paved the way for modern genomics-based discovery approaches.

While natural products are chemically extremely diverse, their biosynthetic machineries are often highly conserved. Core biosynthetic enzymes are characterized by high amino-acid-sequence similarity, which allows screening of genomic data for the presence of specific biosynthetic genes that encode the required enzymatic activity. Eminent examples of genetically programmed molecular assembly lines include the major classes of natural products such as polyketides^4^ and nonribosomally synthesized peptides (NRPs)^5^, ribosomally synthesized and posttranslationally modified peptides (RiPPs)^6^, alkaloids^7^, and terpenes^8^.

Analyses of genome sequences indicate that the biosynthetic potential of bacteria, fungi, and even higher organisms is much larger than what is observed under laboratory conditions. This may either be due to a strong downregulation of the biosynthetic genes or due to low production yields, which prevent detection of the compounds by analytical approaches^9^. To provide access to these compounds, specific triggers or stimuli are required to activate silent or downregulated gene clusters and to increase the compound production rates^10^.

Today, vast genomic data are available and much progress has been made in data mining, compound monitoring, single-cell techniques, and genetic approaches for pathway activation, providing ideal conditions to access the cryptic metabolome.

This review highlights recent advances (since 2015) in the genomics-guided discovery of secondary metabolites focusing on unconventional natural product sources and ecology-inspired discovery approaches.

## Genome mining tools and strategies

**Fig. 1:**
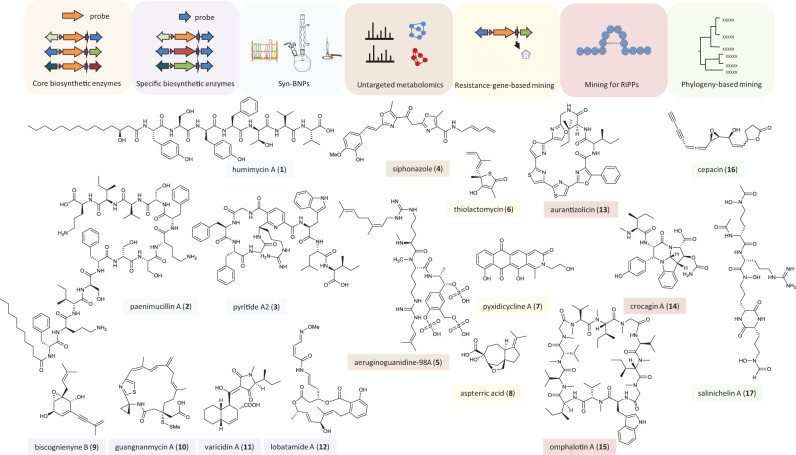
Genome mining strategies. Tools and strategies for mining genomic data and examples of natural products discovered through these methods. (syn-BNP synthetic-bioinformatic natural products, RiPPs ribosomally synthesized and posttranslationally modified peptides). The color code of the boxes around the compound names and icons indicates the strategy that was applied for the discovery of the respective compound: light orange—mining for core biosynthetic enzymes, light violet—mining for specific biosynthetic enzymes, light blue—bioinformatic structure prediction and chemical synthesis, brown—metabolomic approaches to link genes to metabolites and prioritize cryptic BGCs, yellow—resistance-genes-based genome mining, light red—genome mining for RiPPs, green—phylogeny-based genome mining.

The advance of modern sequencing technologies has led to huge amounts of genomic sequence data revealing a tremendous reservoir of likely bioactive natural products that wait to be discovered. As most of the encoded chemical diversity still remains untapped, novel tools and strategies are required to access these potential drug candidates or chemical mediators. Major bottlenecks in realizing this potential include the recognition and prioritization of interesting biosynthetic genes, their activation, and, finally, establishing the link between the genes and the encoded secondary metabolites. To address these challenges, a number of strategies were developed to make use of the acquired genomic information (Fig. 1).

### Automated bioinformatics

The exponential growth of genomic sequencing data has propelled the development of bioinformatic tools to analyze these valuable data. On the basis of our current understanding of the biosynthetic logic, algorithms were created that allow the prediction of natural product biosynthetic assembly lines and putatively encoded structures from gene sequences. Overviews on available automated software tools were provided in a number of comprehensive reviews^11–16^. Most predictive tools rely on homologies to already characterized pathways. Thus, the output is likely biased toward common biosynthetic principles and may fail to detect novel pathways. To overcome this limitation, machine learning-based approaches and deep learning strategies were developed that show an improved ability to identify biosynthetic gene clusters (BGCs) of novel classes^17–21^.

These computational tools proved to be invaluable to mine the huge amount of available genomic information and allow even the nonexpert user to automatically analyze genetic data as a starting point for further experimental investigations.

### Structure prediction and chemical synthesis

Once a BGC of interest has been detected, the next challenge is the link to the corresponding natural product. If the microorganisms are recalcitrant to cultivation or the biosynthetic genes remain silent under laboratory conditions, isolation from large-scale bacterial fermentations is not feasible. As an alternative, a culture-independent approach to access cryptic metabolites was developed based on bioinformatics prediction of chemical scaffolds followed by chemical synthesis of the desired compounds (Fig. 1). This approach bypasses time-consuming activation and isolation procedures and may yield novel chemicals; however, it needs to be taken into account that the predicted structures may only resemble the original metabolite, as post-assembly-line modifications cannot be accurately predicted. Using this method, a number of synthetic-bioinformatic natural products (syn-BNPs) were discovered, including the peptide humimycin (**1**) with potent antimethicillin-resistant *Staphylococcus aureus* (MRSA) activity^22^, the antibiotic paenimucillins (e.g., paenimucillin A, **2**)^23,24^, and an antifungal peptide^23^. Similar methodologies were applied to identify novel RiPPs with antibacterial activity^25^ and a new class of RiPPs, the pyritides^26^. Based on the architecture of a BGC in *Micromonospora rosaria*, the corresponding natural products were predicted to undergo a formal, enzymatic [4 + 2]-cycloaddition with subsequent elimination of the leader peptide and water to produce a pyridine-based macrocycle (pyritide A2, **3**)^26^. Chemical synthesis of the predicted structures and chemo-enzymatic reconstitution of the pathway confirmed the validity of the hypothesis and demonstrated the combined power of bioinformatics and chemical synthesis for investigating cryptic gene clusters. Nonetheless, the application of this strategy is currently limited to compound classes for which accurate prediction algorithms are established (e.g., NRPs and certain RiPPs). For the majority of the cryptic BGCs, bioinformatics software is currently not able to predict the exact compound structures. Combined genomic and metabolomic approaches that employ large-scale mass spectrometry-based comparative metabolomics to predict modifications may subsequently overcome these limitations^27–29^.

### Linking genes to metabolites and prioritizing cryptic BGCs

Various compounds were discovered by traditional approaches like bioactivity-guided isolation in the past, but the molecular bases of their biosynthesis or congeners with other activity profiles remained unknown. Software tools like rBAN^30^ that simulate the retro-biosynthesis of NRPs from their chemical structure can predict the required enzymatic machinery and also help to prioritize promising BGCs for novel compound discovery. In addition, a number of mass spectrometry-guided genome mining approaches were developed that combine genomics and untargeted metabolomics to assign detected secondary metabolites to orphan BGCs and to prioritize strains^29,31–33^ (Fig. 1). An early application of pattern-based genome mining integrating the analysis of BGCs and molecular networking involved the investigation of a large collection of environmental *Salinispora* isolates, which uncovered a huge metabolic diversity among the strains and led to the characterization of novel compounds^32^. Siphonazole (**4**) is an antiplasmodial natural product isolated from a *Herpetosiphon* species. Its biosynthesis has remained elusive for nearly a decade. Through a combination of genome mining, imaging mass spectrometry, and expression studies in the natural producer, the BGC was discovered, revealing that siphonazole originates from a mixed polyketide synthase/nonribosomal peptide synthetase (PKS/NRPS) pathway^34^. Using a similar approach, the cyanobacterial compound aeruginoguanidine (**5**) was linked to a cryptic NRPS gene cluster^35^.

A crucial aspect of genomics-based natural product discovery is the prioritization of the most promising BGCs among the huge number of detected genetic loci. Bioinformatics tools that allow to group related genes by sequence similarity networks^36^, genome neighborhood networks^36,37^, or BGC family^38^ may assist in identifying a specific biosynthetic background. Combining the genomic datasets with automated MS-based metabolomics analysis helps to prioritize novel compounds for structure elucidation^32^. Additionally, target-based genome mining strategies (see below) may accomplish the discovery of natural products with biological/pharmacological potential^39,40^.

### Specialized mining strategies

In most cases, genome mining approaches target core biosynthetic genes of molecular assembly lines.

With the aim to specifically search for compounds with defined bioactivity or with novel structures or even new scaffolds, alternative strategies were established that target, for example, genes encoding resistance information or tailoring enzymes. In addition, a number of phylogeny-guided approaches have been pursued.

#### Resistance genes-based mining

One strategy to specifically search for antibiotic natural products is to mine microbial genomes for resistance genes (Fig. 1). Bacteria have evolved several strategies to avoid the self-toxicity of their antibiotics, including enzyme-catalyzed antibiotic modifications, bypass of antibiotic targets, and active efflux of drugs from the cell^41^. The required resistance genes are often co-localized with the genes encoding the biosynthetic machinery for antibiotic production and can thus serve as a guide to discover putative antibiotics^42^. Mining the genomes of 86 *Salinispora* strains for putative target-modifying resistance genes associated with natural product biosynthetic genes led to the prioritization of an orphan PKS-NRPS hybrid gene cluster harboring a putative fatty acid synthase resistance gene as a candidate for targeted antibiotic discovery. Heterologous expression of the gene cluster in a *Streptomyces* host led to the identification of a group of thiotetronic acid natural products, including the previously known fatty acid synthase inhibitor thiolactomycin (**6**)^43^. Even though the chemical structure of this compound had been long known, this work revealed the molecular basis of thiolactomycin biosynthesis for the first time and demonstrated the feasibility of such an approach. Guided by the presence of genes coding for pentapeptide repeat proteins known for conferring resistance to topoisomerase inhibitors, in the genome of the myxobacterium *Pyxidicoccus fallax*, a cryptic PKS gene cluster was targeted. Its activation in the native host as well as its heterologous expression enabled the structure elucidation of pyxidicyclines (e.g., pyxidicyclin A, **7**)^44^. A similar strategy was successfully applied for the targeted discovery of a novel bioactive compound from filamentous fungi. With the aim to discovering a potential herbicide, published fungal genomes were scanned for genes coding for dihydroxyacid dehydratase (DHAD) that are co-localized with core biosynthetic enzymes. DHAD is an essential enzyme in the indispensable branched-chain amino acid biosynthetic pathway in plants and a common target for herbicides. A homolog of a DHAD encoding gene was identified in the vicinity to genes encoding a sesquiterpene cyclase homolog and two cytochrome P450s in *Aspergillus terreus*. Since this set of genes was highly conserved among a number of fungal genomes, it was hypothesized that it might code for a natural product with DHAD inhibitory activity. Heterologous expression of this gene cluster in *Saccharomyces cerevisiae* and subsequent compound isolation and characterization revealed aspterric acid (**8**) as the encoded natural product and confirmed its DHAD inhibitory activity^45^. These examples demonstrate that genes conferring self-resistance can serve as an indicator of biosynthetic machinery encoding putative antibiotics or toxins. Automated tools to connect genomic and structural information with resistance determinants of known antibiotics will further support resistance-based mining efforts^40,46–48^.

#### Mining for genes encoding specific biosynthetic enzymes (other than canonical PKS and NRPS)

Whereas the majority of genome mining approaches target core biosynthetic enzymes such as canonical PKSs or NRPSs, also genes encoding tailoring enzymes or unusual modules in biosynthetic assembly lines proved to be promising alternatives for mining efforts (Fig. 1). For example, scanning genomic data for sequences of bacterial acetylenases uncovered the biosynthetic machineries for secondary metabolites bearing terminal alkyne moieties^49^. The characterization of the biosynthetic pathway of the acetylenic meroterpenoid biscognienyne B (**9**) allows further genome mining endeavors for the discovery of new compounds with acetylenic prenyl chains^50^.

Using the DUF–SH didomain, responsible for sulfur incorporation in the leinamycin biosynthetic pathway, as a probe to mine for leinamycin analogs, a variety of potential producers of this compound class were discovered (e.g., guangnanmycin A, **10**)^51^. Similar approaches were chosen to discover novel fungal secondary metabolites. Diels-Alderases are a class of enzymes that catalyze pericyclic reactions of a conjugated diene to a dienophile in analogy to a Diels-Alder reaction known from synthetic chemistry. Genes encoding putative Diels-Alderases can be found in various biosynthetic pathways; however, most of the encoded metabolites have remained elusive. Upon mining for genes coding for such enzymes, the BGC for varicidin A (**11**) in *Penicillium variabile* was discovered and the corresponding natural product identified. Varicidin A is a new antifungal natural product containing a *cis*-octahydrodecalin core, biosynthesized by a Diels-Alderase^52^.

Mining genomes for genes encoding noncanonical PKS homologs led to the identification of an architecturally unique *trans*-AT PKS gene cluster in a *Methylobacterium* strain. The gene locus eluded automated prediction due to its unusual and highly fragmented nature. Yet, orthologous clusters could be detected in related species, suggesting that the gene cluster is functional. Comparative screening of culture extracts of a deletion mutant and the wild-type strain uncovered novel polyketides with rare epoxide and cyclopropyl moieties^53^. Similarly, mining for genes encoding oxygenase-containing modules in *trans*-AT PKS systems led to the discovery of the BGC for lobatamide A (**12**) in the culturable plant symbiont *Gynuella sunshinyii*^54^.

#### Mining for ribosomally synthesized peptides

RiPPs are a structurally diverse group of natural products with a wide spectrum of biological activities. Their biosynthesis proceeds via ribosomally assembled precursor peptides that undergo posttranslational modification to gain their biological function^16^. A number of bioinformatics tools were developed to detect the biosynthetic prerequisites in microbial genomes initially relying on core biosynthetic enzymes^55^ (Fig. 1). Later on, class-independent RiPP genome mining tools utilizing alternative probes such as the RiPP recognition elements (RRE) were established^16,25,55–58^. Whereas potential producers of RiPPs can thus be identified through comparative genome mining, additional methods are required to actually find the corresponding metabolite. Software tools that integrate genomic and metabolomic data may additionally support the identification of novel RiPPs^27,28,58^. For example, bioinformatics prediction using RiPP-PRISM in tandem with automated LC-MS/MS searches led to the identification of aurantizolicin (**13**)^59^. Through a combination of data mining and analytical chemistry, crocagins (e.g., crocagin A, **14**) were discovered from the myxobacterium *Chondromyces crocatus* that form a new class of RiPPs^60^. Polytheonamides are the only characterized members of a unique family of RiPPs termed proteusins (named after the Greek sea god Proteus constantly changing his shape), based on an unusually large leader peptide with homology to nitrile hydratases. These marine sponge-derived peptides are chemically distinct from any other known natural product. Their ribosomal precursor peptide undergoes 49 mostly noncanonical posttranslational modifications, which results in a highly cytotoxic natural product. As the original producer of these hypermodified peptides cannot be cultivated, an alternative producing platform was required. Data mining revealed that closely related pathways are present in taxonomically and ecologically remarkably diverse organisms, including culturable bacteria. Using one candidate species as a host, a platform was established that allows the production of highly modified polytheonamide-like peptides with cytostatic properties^61^.

While major progress has been made in understanding the biosynthesis of RiPPs in bacteria, only little is known about the formation of ribosomal peptides in fungi. One example of a RiPP from a fungus is omphalotin (**15**), a cyclopeptide with multiple *N*-methylations. Mining the genome of *Omphalotus olearius* for genes encoding a precursor peptide resulted in the identification of a novel biosynthesis mechanism for a RiPP. An iterative *N*-methyltransferase fused to its peptide substrate catalyzes the auto-methylation of its C-terminus^62,63^. Due to this unusual mechanism, the term “borosins” was proposed for this novel RiPP family, referring to the ancient mythological symbol Ouroboros depicting a serpent biting its own tail^63^. Later on, additional members of the borosin class were discovered^64^.

#### Phylogeny-based mining

Combining classical genome mining with evolutionary aspects can further support bioprospecting and may also facilitate functional predictions of biosynthetic genes^38,65^ (Fig. 1). The phylogenies of natural product-producing organisms can be applied to infer talented producers. For example, many members of the genus *Burkholderia* are known to produce a high number of antimicrobial agents and are therefore regarded as potential biocontrol organisms. However, at the same time, *Burkholderia* species are also known to infect humans, which hampers their potential application for biocontrol purposes. Phylogeny-led genome mining in combination with chemical and biological profiling revealed the efficacy of *Burkholderia ambifaria* as a biopesticide. Biosynthesis of the acetylenic antibiotic cepacin (**16**) was shown to be responsible for the pesticidal activity. Deletion of a nonessential plasmid associated with virulence resulted in a less infectious mutant with retained pesticidal activity^66^.

Additionally, studying the evolutionary history of secondary metabolite gene clusters by phylogeny-based methods can also expedite the discovery of novel molecules. Using a strategy called EvoMining, which is based on the assumption that most enzymes from secondary metabolism evolved from primary metabolism, the evolutionary history of 23 enzyme families was reviewed, which led to the discovery of arseno-organic metabolites in *Streptomyces* species^67^. Through the reconstruction of the evolutionary history of two different siderophore families, it was shown that certain *Salinispora* strains have functionally replaced an ancient desferrioxamine pathway and acquired the genetic accessories for the biosynthesis of the novel siderophore salinichelin (**17**)^68^.

## Accessing silent biosynthetic genes

**Fig. 2:**
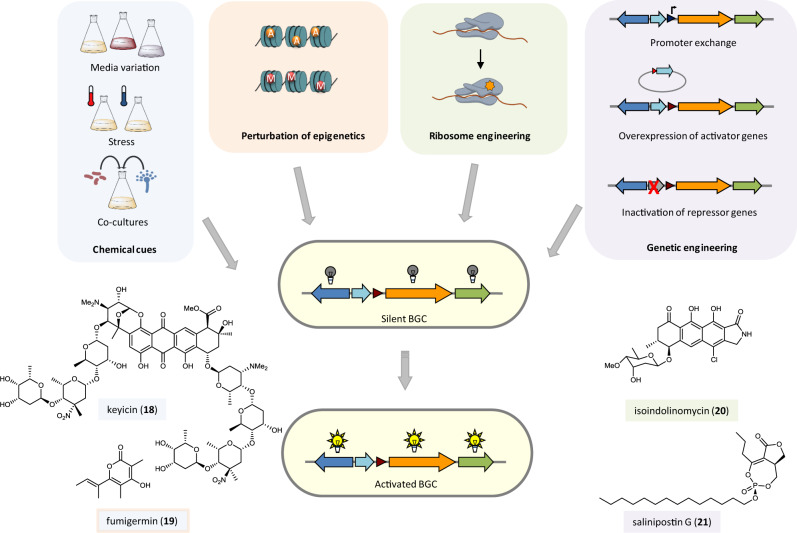
Accessing cryptic biosynthetic genes in native hosts. Strategies for the activation of silent biosynthetic gene clusters in native hosts and examples of natural products discovered through these methods. (BGC biosynthetic gene cluster). The color code of the boxes around the compound names and icons indicates the strategy that was applied for the discovery of the respective compound: light blue—triggering natural product biosynthesis through chemical cues, light orange—activation of natural product biosynthesis through perturbation of epigenetic regulation, green—activation of natural product biosynthesis through ribosome engineering, light violet—activation of natural product biosynthesis through genetic engineering.

**Fig. 3:**
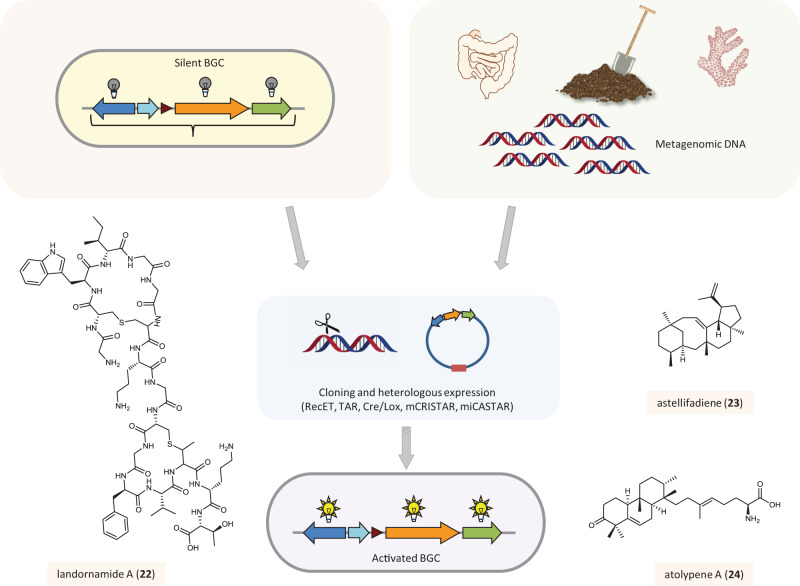
Accessing cryptic biosynthetic genes in heterologous hosts. Strategies for the activation of silent biosynthetic gene clusters in heterologous hosts and examples of natural products discovered through these methods. (BGC biosynthetic gene cluster). The color code of the boxes around the compound names and icons indicates the strategy that was applied for the discovery of the respective compound: Activation of natural product biosynthesis through heterologous expression of biosynthetic genes that are downregulated in the native producer (light orange) or from metagenomic DNA (green).

The finely tuned regulation of secondary metabolism poses a huge challenge to natural product researchers to identify conditions under which biosynthetic genes are expressed. In many cases, biosynthesis is downregulated, and the encoded structures escape detection. Therefore, efforts are required to induce the expression of silent genes and to link chemical structures to orphan biosynthesis gene clusters (Fig. 2 and 3).

### Triggering natural product biosynthesis

The production of secondary metabolites by microorganisms critically depends on the cultivation conditions. Often specific triggers (e.g., small molecules) are required to elicit the expression of the biosynthetic genes (Fig. 2). A systematic variation of culture conditions and/or the application of stress conditions can be an initial approach to propagate the formation of the chemical compounds^10^. This rather empirical method is likely impractical for a large number of different microbes. The ecological background of the potential producers may inspire alternative approaches. Based on the hypothesis that natural products serve as chemical mediators of microbial interaction^2^, several co-culturing methods were developed with the aim that one organism induces the formation of silent metabolites in the other. For example, co-culturing of the marine invertebrate-associated bacteria *Micromonospora* sp. and *Rhodococcus* sp. afforded the polynitroglycosylated anthracycline keyicin (**18**) which is hypothesized to play a role in microbial communication^69^. Likewise, *Streptomyces rapamycinicus* was shown to induce the formation of fumigermin (**19**) in *Aspergillus fumigatus*. Fumigermin resembles bacterial germination inhibitors such as germicidin and, ultimately, its inhibitory activity on spore germination of *S. rapamycinicus* was demonstrated. This study represents one of the rare examples where it could be demonstrated that a compound whose production was elicited in a mixed culture plays a role in the interaction of the co-cultured partners^70^.

Although microbial co-cultivation has proven successful in inducing secondary metabolite biosynthesis, it is still an arbitrary approach that suffers from low predictability and difficult up-scaling possibilities to increase throughput. Moreover, in the majority of cases, the nature of the elicitor remains unknown. To overcome some of these obstacles, a strategy was developed to screen more systematically for inducers of specific silent biosynthetic pathways (HiTES = high-throughput elicitor screens)^71^. This method is based on the assumption that microbes employ small natural products for communication, which may function as elicitors of silent biosynthetic genes. A reporter gene is inserted inside the gene cluster of interest, and the resulting mutant is screened against libraries of secondary metabolites in a high-throughput fashion to find potential inducers. Using this methodology, silent gene clusters in *Burkholderia thailandensis* and *Streptomyces albus* were induced in a targeted fashion and several antibiotic and cytotoxic compounds were identified as potential elicitors of other cryptic biosynthetic pathways^71,72^. In another study, screening of activation conditions was performed with a reporter-guided mutant selection strategy after genome-scale random mutagenesis^73^.

### Activation of silent pathways through ribosome engineering

Ribosome engineering is based on the isolation of spontaneously developed drug-resistant mutants. Through the application of the antibiotics streptomycin or rifampicin, strains with mutations in the *rpsL* gene (encoding the ribosomal protein S12) or *rpoB* gene (encoding the RNA polymerase (RNAP) β-subunit) are selected. Such mutants may show an altered gene expression, which may result in a different metabolite profile. Initially developed in streptomycetes^74^, the method has now been applied to randomly activate silent biosynthetic genes in various strains^75,76^ (Fig. 2). A recent example is the discovery of the polyketide isoindolinomycin through the screening of rifampicin-resistant mutants^77^.

### Genetic approaches to activate silent pathways

While some silent BGCs can be activated in the native host after identification of the appropriate cues, some others require genetic manipulation to induce gene expression, for example, the overexpression of regulatory genes or the introduction of promoters (Fig. 2). In a recent example, comparative transcriptomics was used to identify key regulatory genes of silent pathways. Comparing the expression profiles of similar gene clusters in different strains helped to prioritize producer strains and led to the identification of a series of novel compounds (e.g., salinipostin G, **21**)^78^. A CRISPR-Cas9-based promoter knock-in strategy was applied to activate multiple BGCs in *Streptomyces* species^79^. Whereas methods to engineer microbial pathways are well established for model organisms, the targeted genetic manipulation of nonmodel strains may be highly challenging. To facilitate in situ promoter insertion in strains less amenable to genetic manipulation, such as environmental *Burkholderia* isolates, a strategy was reported that involves novel bacteriophage recombinases (Red αβ homologs). The recombinase genes were cloned for transient expression, and optimized for the efficient deletion of chromosomal DNA. The presented workflow allows targeted gene deletions or promoter knock-ins in various Burkholderiales that lack native Red αβ recombinase homologs^80^.

In eukaryotes, many secondary metabolite gene clusters are located in heterochromatic regions of the genome. Gene transcription is controlled by epigenetic regulation such as histone deacetylation or DNA methylation. Manipulation of epigenetic control may lead to activation of silent biosynthetic genes^81^. Deletion of a histone H3 deacetylase resulted in the pleiotropic activation and overexpression of more than 75% of the biosynthetic genes of the endophytic fungus *Calcarisporium arbuscular*^82^. Modification of the chromatin landscape may also be a strategy that bacteria apply to influence gene transcription in fungi. In *A. nidulans*, changes in histone acetylation were monitored upon co-cultivation with *S. rapamycinicus* and related to changes in the fungal transcriptome^83^.

In strains that are less amenable to genetic manipulation or cannot be cultivated, functional expression of full gene clusters in heterologous hosts may be a feasible alternative (Fig. 3). The prerequisite is the direct capture of the gene cluster by homologous recombination using, for example, the Lambda Red/ET recombination, yeast-based transformation-associated recombination system, or alternative techniques^84–86^. These technologies together with optimized heterologous hosts were successfully applied for the discovery of novel natural products^86,87^.

Reconstruction of a silent RiPP pathway in *E. coli* led to the identification of a new antiviral peptide, landornamide A (**22**), revealing at the same time various biosynthetic novelties^88^. Mining the genome of *Emericella variecolor* NBRC 32302 for prenyl transferase and terpene cyclase encoding genes and functional expression of the identified terpene synthesis genes in *Aspergillus oryzae* afforded the sesterterpene astellifadiene (**23**)^89^.

The introduction of the CRISPR/Cas9 system also offered new possibilities for the refactoring of BGCs^87^. For example, a yeast-based promoter engineering platform was developed that combines CRISPR/Cas9 and transformation-associated recombination to enable single-marker multiplexed promoter engineering of large gene clusters (mCRISTAR)^90^. The target gene cluster is cut at the native promoter regions using CRISPR/Cas9 to allow the TAR-mediated reassembly to incorporate synthetic promoters. Further development of this strategy resulted in a simplified method (miCASTAR) that allows the targeted activation of BGCs, as was demonstrated by the discovery of the sesterterpene atolypene (**24**)^91^.

## Genome-guided discovery of natural products from unconventional sources

**Fig. 4:**
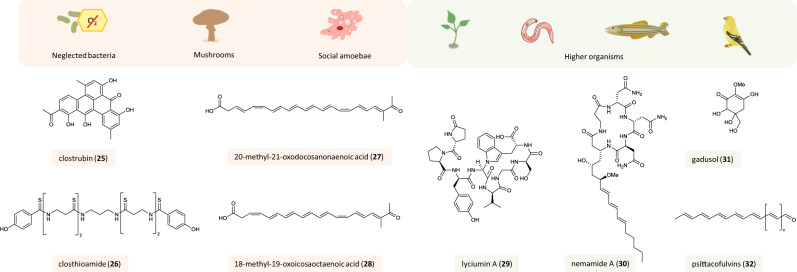
Genome-guided discovery of natural products from unconventional sources. Neglected bacteria, mushrooms, social amoebae, and higher organisms as sources and examples of natural products discovered from these organisms. The color code of the boxes around the compound names and icons indicates the source of the respective compound: light orange—microbes (neglected bacteria, mushrooms, and amoebae), green—higher organisms (plants, nematodes, fishes, birds).

As the search for new natural products from traditional bacterial sources like actinomycetes often results in a high rediscovery rate of known compounds, alternative sources are being explored. The high-throughput sequencing of microorganisms from diverse habitats and genome sequences of higher organisms revealed a huge number of “talented producers” from so far underexplored genera (Fig. 4).

### Genome-guided discovery of natural products from neglected bacteria and archaea

Anaerobic bacteria have long been neglected as a potential source of bioactive secondary metabolites. Genomic analyses indicated that these organisms harbor a huge biosynthetic potential that remains to be discovered^92^. Only recently, a handful of secondary metabolites could be identified through combined genomic and analytical approaches^93–97^ (Fig. 4). Mining the genome of *Clostridium puniceum*, an anaerobic plant pathogen causing potato rot, revealed a gene locus coding for the biosynthesis of the pentacyclic polyketides clostrubins (e.g., clostrubin A, **25**) that are essential for the anaerobic bacteria to grow under aerobic/oxic conditions in association with plants. Moreover, clostrubins were found to possess strong antibiotic activity against major potato pathogens, implying that these metabolites play an important role in niche defense^98^. Another potent antibiotic from a *Clostridium* species (*Ruminiclostridium cellulolyticum*) is closthioamide (CTA, **26**)^99^. Its biosynthesis, however, has remained enigmatic until recently. Now it was demonstrated that CTA is biosynthesized via a novel thiotemplated peptide assembly line that differs from canonical NRPSs and therefore escaped previous genomic analyses with automated software tools^100,101^. This example showcases the limitations of automated genome mining efforts relying on characterized biosynthetic mechanisms.

Archaea are known to generally have small genomes, and analyses of their secondary metabolite BGCs are scarce. A systematic screening of archaea genome sequences for the presence of putative secondary metabolite BGCs revealed that the majority of archaeal genomes only harbor one or two types of secondary metabolite BGCs, with bacteriocin- and terpene-encoding genes being most abundant^102^. NRPS encoding genes are sporadically found^103^. This biosynthetic potential is reflected in the so-far-characterized secondary metabolites^104^.

### Genome-guided discovery of natural products from mushrooms and amoebae

The majority of characterized secondary metabolites from Basidiomycota constitute terpenoids. Genome analyses, however, indicate that mushrooms can synthesize chemically diverse metabolites (Fig. 4). The first reducing PKS from a mushroom was characterized from the stereaceous mushroom BY1. The PKS PPS1 is highly upregulated upon mycelial damage and synthesizes anti-larval polyenes (**27**, **28**), as was shown by heterologous expression in an *Aspergillus* host^105^.

Bioinformatics studies also revealed the presence of a multitude of biosynthetic genes in various social amoebae, qualifying them as a promising source for genomics-based natural product discovery^106^ (Fig. 4). Genome mining revealed the presence of classical terpene synthases in six species of amoebae. Functional expression in *E. coli* and metabolic profiling of amoebae cultures demonstrated that these organisms are able to produce a variety of terpenes. The fact that the production is restricted to specific periods during multicellular development suggests a functional role for these compounds in the native habitat or the life cycle^107^.

### Genome-guided discovery of natural products from higher organisms

Plant-derived natural products have been appreciated as medicinal agents for a long time, but genomics-guided approaches to discover novel secondary metabolites have been pursued only since the advent of modern sequencing technologies^108^ (Fig. 4). Nowadays prediction and characterization of entire pathways are possible. Limonoids are triterpenes contributing to the bitter taste of citrus fruits and are well known for their insecticidal activity and their potential pharmaceutical properties. Through mining the genomes and transcriptomes of three diverse limonoid-producing species and expression studies, the first insight into the biosynthesis of these triterpenes was gained^109^. Likewise, targeted mining of available plant genome sequences revealed co-localized prenyl transferase and terpene synthase genes for the biosynthesis of sesterterpenes in the Brassicaceae family. Expression of these genes in *Nicotiana benthamiae* resulted in the formation of fungal-type sesterterpenes^110^. Analysis of the transcriptome data of Chinese wolfberry (*Lycium chinense*) using the predicted core peptide sequences of three lyciumin isoforms as a probe uncovered the molecular basis of RiPP biosynthesis in plants. The lyciumin precursor gene LbaLycA was identified from *L. barbarum* and then characterized by heterologous expression in tobacco leaves to confirm its role in lyciumin (**29**) biosynthesis^111^.

Whereas PKS and NRPS gene clusters are commonly found in many bacterial and fungal genomes, they are underrepresented in animals. Yet, the genome of the model organism *Caenorhabditis elegans* harbors a huge, multi-module hybrid PKS/NRPS and a large multi-module NRPS. To identify the encoded products, LC-MS based, comparative untargeted metabolomics of wild-type and deletion mutants was performed, which resulted in the identification of nemamides (e.g., nemamide A, **30**), which are important for larval development and represent the first complex PKS-NRPS hybrid metabolites from a metazoan^112^ (Fig. 4).

In another study, it was found that identical metabolites may be synthesized by microbes and animals via different pathways. Mycosporine-like amino acids and gadusols are UV-vis protective compounds produced by different marine microorganisms. They are also found in corals, marine invertebrates, and fish, and it was hypothesized that marine higher organisms obtain these compounds exclusively from their diet. However, by genome mining, it was found that zebrafish harbor putative gadusol-encoding genes. Expression analysis in the native producer and metabolic profiling of zebrafish embryos demonstrated that this organism actually synthesizes gadusol (**31**) de novo. Using the identified genes as an in silico probe, gadusol biosynthetic genes could also be detected in the genomes of birds, reptiles, and other organisms, raising the question of how this BGC evolved^113^ (Fig. 4).

Recently, the first functional evidence for a PKS in vertebrates was gained. In contrast to the majority of birds where red and yellow colors of the feathers are derived from carotenoid pigments obtained from the diet, parrots employ a pigment called psittacofulvin (**32**). Through genome mining, association mapping, and gene expression analysis, the gene responsible for the yellow pigmentation in the feathers of budgerigars was identified. Psittacofulvin pigments are synthesized by the PKS gene *MuPKS*, a pre-existing gene co-opted into developing feathers. Interestingly, homologous genes were identified in other birds^114^ (Fig. 4).

## Genome-guided discovery of natural products from ecological interactions

**Fig. 5:**
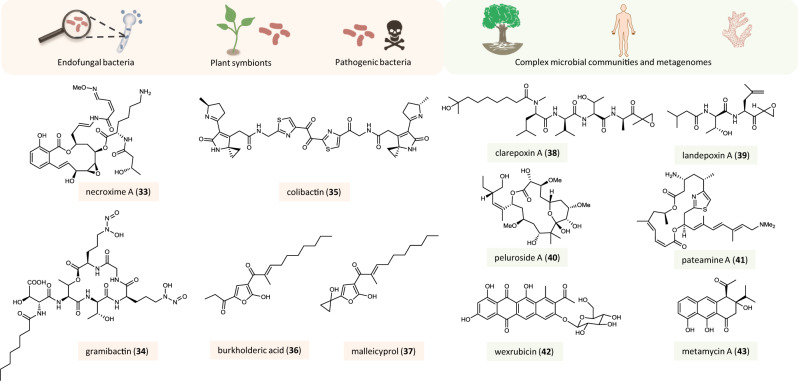
Genome-guided discovery of natural products from ecological interactions. Symbiotic relationships among few or multiple partners as sources and examples of natural products discovered from these interactions. The color code of the boxes around the compound names and icons indicates the source of the respective compound: light orange—interactions among defined partners (endofungal bacteria, plant symbionts, and pathogenic bacteria), green—interactions among complex microbial communities (plant and human microbiota as well as marine sponges).

Secondary metabolites play important roles as mediators of interactions among different organisms. Therefore, taking the ecological context into account can support the discovery of new bioactive compounds^115^ (Fig. 5).

### Genome-guided discovery of natural products from symbiotic bacteria

Microorganisms involved in symbiotic relationships with higher organisms have been increasingly recognized as a promising source for genomics-driven natural product discovery^116^ (Fig. 5). For example, by a combination of genome mining and chemical analytics, a number of nonribosomally synthesized peptides were identified from the endosymbiont of the plant-pathogenic fungus *Rhizopus microsporus* that are involved in the bacterial–fungal interaction and/or serve an ecological function^117–119^. The endofungal bacteria also produce cytotoxic necroximes (e.g., necroxime A, **33**) in symbiosis with the fungal host. These benzolactones are biosynthesized by a modular PKS/NRPS assembly line and may contribute to the pathogenic phenotypes ascribed to the fungal host^120^. A genome-guided chemical profiling of a marine bacterium from the rhizosphere of the halophilic plant *Carex scabrifolia* yielded a number of chemically diverse natural products, some of which possess potent cytostatic activity^121^. A novel type of siderophore featuring diazeniumdiolate moieties for iron binding (gramibactin, **34**) was identified from the rhizosphere-associated strain *Paraburkholderia graminis*. As the corresponding gene locus is highly conserved in numerous other plant-associated bacteria, it was hypothesized that gramibactin may solubilize iron to make it accessible to the plant^122^. Subsequently, genome mining identified other types of diazeniumdiolate siderophores from other *Burkholderia* species^123^.

### Mining genomes of pathogenic microorganisms

In many cases, natural products serve as virulence factors in pathogenic interactions^2^. Targeting the cryptic metabolome of pathogenic microorganisms (Fig. 5) may not only contribute to the understanding of pathogenicity mechanisms, but also open new opportunities to combat diseases. A salient example for the genome-based discovery of a virulence factor is the cytotoxin colibactin (**35**). From the initial discovery of its biosynthetic locus in the genome of human gut bacteria, it took more than a decade and joint forces of several workgroups until its chemical structure was elucidated^124,125^. Another example concerns the infectious diseases glanders and melioidosis caused by *Burkholderia* species. Through targeted promoter exchange a cryptic NRPS/PKS hybrid gene cluster had been activated, which led to the identification of burkholderic acid (**36**, *syn*. malleilactone) in the pathogens *B. thailandensis*/*B. mallei*^126,127^. Since this metabolite did not account for the observed pathogenic phenotype, additional compounds of this gene cluster were identified in a follow-up study. By metabolic profiling and molecular network analyses of the model organism *B. thailandensis*, unusual cyclopropanol-substituted polyketides (e.g., malleicyprol, **37**) were identified as the primary products of the cryptic pathway and shown to be highly active in a nematode infection model^128^.

### Mining microbiomes and metagenomes

The majority of the bacterial diversity in the environment has remained undetected due to the limitations of culturing techniques. Rapid and inexpensive sequencing technologies and bioinformatics mining of the acquired sequencing data have allowed a glimpse of the wealth of the encoded chemical diversity. It has become apparent that complex microbial communities, as encountered for example in the soil, the marine environment, or in animals/humans, are a promising source of novel natural products. Metagenomics approaches may bypass cultivation or expression problems and provide access to these molecules (Fig. 5).

For example, a metagenome exploration of *Dysideidae* sponges resulted in the identification of the BGCs responsible for the formation of cytotoxic polybrominated diphenyl ethers. The functionality of the biosynthetic genes was experimentally proven by heterologous expression in a cyanobacterial host and the origin of the compounds from the primary cyanobacterial symbiont *Hormoscilla spongeliae* was demonstrated^129^. Recovering environmental DNA sequences related to known epoxyketone biosynthesis genes and heterologous expression of the complete gene clusters led to the identification of the epoxyketone proteasome inhibitors clarepoxcin A (**38**) and landepoxcin A (**39**)^130^. The sponge *Mycale hentscheli* is known for the production of highly active secondary metabolites such as the microtubule-stabilizing pelorusides (e.g., peluroside A, **40**), mycalamide-type contact poisons, and the pateamines (e.g., pateamine A, **41**) with anticancer activity. Investigations of the sponge microbiome established the biosynthetic background of these potent natural products and revealed that in contrast to other sponges with known ‘super-producer’ symbionts, the huge chemical diversity is likely created by multiple bacterial species^131,132^.

A systematic analysis of the microbiome associated with the model plant *Arabidopsis thaliana* demonstrated the huge biosynthetic potential of its symbionts. Through a high-throughput interaction screening system of a strain collection of more than 200 leaf isolates and genome mining, more than 1000 BGCs for compounds with a possible ecological relevance were identified. To demonstrate the rationality of this approach, several bioactive metabolites were characterized from a selected bacterium by bioactivity- and genomics-guided approaches^133^.

The human microbiota have also been recognized as a fruitful source for novel metabolites, especially for antibiotics^134–136^. The growing availability of human microbiome sequence data has spurred efforts to develop efficient systems to leverage the encoded diversity. Assemblies of complex metagenomic sequencing data frequently consist of fragmented BGCs and sequences of the most abundant members of the microbiome are overrepresented. Thus, the biosynthetic capabilities of less abundant bacteria often remain hidden. Therefore, an assembly-independent approach was developed that allows the direct identification of BGCs from metagenomics reads. With this method, a huge human microbiome data set was analyzed, which resulted in the identification of type II PKS gene clusters that are widely distributed among gut, oral, and skin microbiomes. Cloning and heterologous expression of selected genes led to the discovery of wexrubicin (**42**) and metamycins (e.g., metamycin A, **43**) with antibiotic activities^137^. Techniques of single-cell genomics are currently being explored by natural product researchers as an alternative option to address these problems^138^.

## Conclusions and Perspectives

Advances in genomics and bioinformatics have reinvigorated natural product research to become a more targeted and systematic research endeavor with the genomic information as the starting point. Rapid and inexpensive sequencing technologies along with powerful bioinformatics tools have furthered our insights into microbial and structural diversity, revealing nearly unlimited natural product discovery potential. Besides the targeted genome-guided reinvestigation of well-known producers, the exploration of unconventional sources such as anaerobic bacteria or higher organisms promises exciting discoveries. Likewise, the investigation of natural products in their native environment (as mediators of pairwise or complex organismal interaction) will not only help to understand natural product functions, but also uncover novel drug candidates inspired from the ecological function of secondary metabolites. Major hurdles in accessing the hidden chemical diversity will be subsequently overcome by the ongoing development of innovative culturing methods, efficient genome editing, and optimized expression systems. Along with more sensitive chemical analytics, this will especially apply leverage to the discovery of novel metabolites from metagenomics data. Single-cell-based technologies will open additional possibilities to study biosynthetic capabilities of individual members of microbiomes.

## Data Availability

Data sharing not applicable as no original research is reported.
